# Association of *CD58* Polymorphism with Multiple Sclerosis and Response to Interferon ß Therapy in A Subset of Iranian Population

**DOI:** 10.22074/cellj.2015.505

**Published:** 2015-01-13

**Authors:** Sara Torbati, Fatemeh Karami, Majid Ghaffarpour, Mahdi Zamani

**Affiliations:** 1 Department of Neurogenetics, Iranian Center of Neurological Research, Tehran University of Medical Sciences, Tehran, Iran; 2 Department of Medical Genetics, School of Medicine, Tehran University of Medical Sciences, Tehran, Iran

**Keywords:** Multiple Sclerosis, CD58, Polymorphism, Interferon β, Response

## Abstract

**Objective:**

Multiple sclerosis (MS) is one of the leading neurodegenerative causes of physical disability world-wide. Genetic aberrations of autoimmunity pathway components have been demonstrated to significantly influence MS development. *Cluster of Differentiation 58 (CD58)* is pertained to a group of genes which had been assayed in several recent association studies. Given the significance of *CD58* in modulation of T regulatory cells that control autoimmune responses, the present study was conducted to investigate the frequency of rs12044852 polymorphism and its effect on the outcome of interferon beta (IFN-β) therapy in a subset of Iranian MS patients.

**Materials and Methods:**

Two hundred MS patients and equal number of healthy controls were recruited to be genotyped in an experimental case-control based study through polymerase chain reaction using specific sequence primers (PCR-SSP). Relapsing remitting multiple sclerosis (RRMS) patients administered IFN-β therapy were followed up with clinical visits every three months up to two years. The mean of multiple sclerosis severity score (MSSS) and expanded disability status scale (EDSS) were measured to monitor the change in severity of MS in response to IFN-β therapy. Pearson’s Chi-square and analysis of variance (ANOVA) tests were the main statistical methods used in this study.

**Results:**

Strong association was found between the CC genotype and onset of MS (p=0.001, OR=2.22). However, there was no association between rs12044852 and various classifications and severity of MS. Pharmacogenetics-based analysis indicated that carriers of CC genotype had the highest MSSS score compared to others, implying a negative impact of rs12044852 on response to IFN-β therapy.

**Conclusion:**

Taken together, our findings revealed the critical effect of rs12044852 polymorphism of *CD58* on the progression of MS disease. This indicates that genotyping of MS patients may expedite achieving personalized medical management of MS patients.

## Introduction

Multiple sclerosis (MS) is an autoimmune and neurodegenerative disorder in which the myelin membrane is attacked by immune system leading to focal nervous system lesions, axonal loss and brain atrophy. It affects 41.8 and 69.1 per 100,000 person-years among Iranian and worldwide populations respectively (1, 2). MS patients usually die as a corollary of disease complications with mortality rate of 1 per 1,000 subjects. Viral infections, ethnicity, deprivation of sun exposure and vitamin D deficiency are some of the most important geographical features and immunologic factors proposed for MS etiology. Based on the location of produced inflammation and demyelization, there are five main types of MS which are associated with different presentations including primary-progressive multiple sclerosis (PPMS), relapsingremitting multiple sclerosis (RRMS), secondary- progressive multiple sclerosis (SPMS) and progressive-relapsing multiple sclerosis (PRMS) and clinically isolated syndrome (CIS) (3). Familial aggregation, higher risk of disease in first, second and third degree of relatives, and consanguineous marriages in addition to the results of adoption studies are consistent with a significant role of genetic factors in pathogenesis of MS. Several mechanisms have been suggested for central nervous system (CNS) lesions such as reduced expression of mitochondrial genes and autoimmunity as a result of aberrant T cells selections (4). Normal T lymphocyte selection during thymus maturation may be influenced by polymorphisms within HLA genes comprising close to half percent of the genetic contribution to MS. HLA- DRB1*1501- DQB1*0602 haplotype, related to HLA-DR15 locus, is the most important genetic risk factor identified in various MS studies with odds ratios (OR) of 2-3.5 (5, 6). However, polymorphisms in the remaining proportion of heritable genes may also be associated with generation of autoreactive T cells. These include *Cluster of Differentiation 226 (CD226), C-type lectin domain family 16, member A (CLEC16-A), interleukin 2 receptor alpha (IL2-RA), IL7-RA* and *CD58* for which their association was found to be significant in various genome wide association studies (GWAS) (7). The *CD58* gene encodes for a glycosylated cell adhesion protein called lymphocyte function-associated antigen 3 (LFA3) which was mapped on human chromosome 1p13. It is expressed on antigen presenting cells (APCs) particularly macrophages and enhance the APCs attachment to T cells through binding to its specific ligand (CD2 or LFA2) present on T cell surface (8, 9). Murine studies have shown that CD58-CD2 interaction has a central role in antigen recognition and both positive and negative selection of T cells (10). Therefore, *CD58* is a potential target for examining the association of its genetic variants with autoimmunity within the context of MS pathogenesis. The role of *CD58* gene polymorphisms was corroborated in development or protection against MS in different human studies (11).

Herein, we are aimed to determine the frequency of alleles and genotypes regarding the intronic rs12044852 polymorphism (g.117087779C>A) of *CD58* in a subset of Iranian MS patients (RRMS, PPMS, SPMS and CIS types) and the healthy control group. The rs12044852 polymorphism is one of the most important variants of *CD58* which was found to have significant association with MS disease (11). Given the effect of interferon β (IFN- β) on *CD58* expression (12), we also conducted a prospective case-only study to assess the effect of the mentioned variant on the therapeutic response of MS patients to IFN- β therapy followed for two years.

## Materials and Methods

### Sample selection

Two hundred unrelated MS patients who were diagnosed by neurologists according to the revised criteria (Mc Donald, 2010) (13) were enrolled in the present case-control study. The MS subjects were chosen from patients admitted to neurologic center of Imam Khomeini Hospital Complex within 2011-2012. The same number of healthy controls matched for age (17-70 years old for cases and 13-65 years old for controls) and gender were recruited from the staff of the same hospital to be genotyped and compared with MS patients in a case-control designed study. The number of cases and controls were determined through the sample size formula (n=2 (z1- α +z1- β) 2 pq/(P1-p0) 2) and the frequency of minor allele A was defined as 0.11 based on a previous report (14).

Of note, none of the controls and their families had previous history of MS. Patients who had any other autoimmune disease or genetic disorders were excluded from our study. An informative form was filled in for every patient containing type of MS, age of onset of disease, duration of interval time between first attack and first relapse, number of relapse episode in recurrent types and drug history. Demographic and clinical data of MS patients are represented in table 1. The severity of MS disease was scored by using the multiple sclerosis severity score (MSSS) criteria.

**Table 1:** Demographic and clinical characteristics of MS patients


Characteristics of Ms patients	Findings

**Age (Y) mean ± SD**	35.3 ± 9.73
**Female/male (n)**	138/62 (69%/31%)
**Age of onset (Y) mean ± SD**	28.8 ± 8.60
**Disease duration (Y) mean ± SD**	6.7 ± 5.37
**Positive family history**	13(6.5%)
**Negative family history**	187 (93.5%)
**Positive history of autoimmune disease**	0 (0%)
**Negative history of autoimmune disease**	200 (100%)
**EDSS mean ± SD**	2.8 ± 2.09
**MSSS mean ± SD**	4.8 ± 2.90
**MS Subgroups (n)**	
**RRMS**	132 (66%)
**SPMS**	27(13.5%)
**PPMS**	20(10%)
**CIS**	21(10.5%)
**Types of given interferon β**	
**IFNβ-1a IM**	76
**IFNβ-1a SC**	30
**IFNβ-1b SC**	14


MS; Multiple sclerosis, SD; Standard deviation, EDSS; Expanded disability status scale, MSSS; Multiple sclerosis se-verity score, RRMS; Relapsing remitting multiple sclerosis, SPMS; Secondary-progressive multiple sclerosis, PPMS; Primary-progressive multiple sclerosis, CIS; Clinically isolated syndrome, IFN; Interferon, IM; Intramuscular and SC; Subcutaneous.

### DNA isolation

Five mL of peripheral blood was obtained from both case and control groups in canonical tubes containing ethylene diamine tetra acetic acid (EDTA) as anticoagulant for blood. Genomic DNA was isolated from blood samples using DNA extraction Blood Mini Kit (Qiagen, Chatsworth, CA). The quality, purity and quantity of isolated DNA samples were determined using NanoDrop ND-1000 spectrophotometer (NanoDrop Technologies, Wilmington, DE) and electrophoresis on a 1% agarose gel.

### Polymerase chain reaction by specific sequence primers (PCR-SSP)

The rs12044852 A/C polymorphism in CD58 was genotyped by PCR-SSP. The primer pair harnessed for amplification of each DNA template was allele specific and designed through online primer 3 program and were as following: 5'CACACGTGATTCCTAACATC 3' as forward for normal allele, 5' CACACGTGATTCCTAACATA 3' for mutant allele and 5' CCGCTCTCTACTCTAAAGAC 3' as the common reverse primer. The PCR mixture included 10 pmol of each forward and reverse primers, 2.5 μl of 10x buffer including 1.5 mM Mgcl_2_, 0.2 mM of dNTP mixture and 1U of Taq DNA polymerase (Cynagen, Iran) in addition to 100 ng of each genomic DNA sample adjusted with ddH_2_O up to final volume of 25 μl. After an initial denaturation at 94.C for 5 minutes, 31 cycles of PCR was performed according to the following program in a Biorad thermocycler (Bio-Rad Laboratories, Hercules, CA, USA): denaturation at 94.C for 30 seconds, annealing at 50.C for 30 seconds, extension at 72.C for 60 second and final extension at 72.C for 10 minutes. The PCR products were resolved on polyacrylamide gel electrophoresis stained by SYBR. Green I nucleic acid gel stain and the size of PCR product was 219 bps. Different genotypes of rs12044852 A/C polymorphism were identified on a non-denaturing polyacrylamide gel (10%) by presence or absence of PCR products.

### Case-only study

To investigate the association between rs12044852 polymorphism of CD58 and drug response in MS patients, a subset of our MS patients were given IFN-β and followed for two years. In this regard, all the genotyped RRMS patients who had undergone IFN-β therapy were physically examined every three months up to two years to assess the effect of treatment on constant changes in severity of disease. In addition, a new expanded disability status scale (EDSS) and MSSS scores was determined for each RRMS patient in every visit. It is noteworthy that MSSS score makes correlation between scores obtained for EDSS and the distribution of disabilities affecting various MS patients with different disease durations (15). IFN-β non-responder status was defined according to criteria previously reported (16). Relapse is characterized when one or more neurologic complication takes longer than 24 hours as confirmed by a neurologist’s examination.

### Statistical analysis

Fisher’s exact and Chi-square tests were used for comparing the frequency of genotypes and alleles of rs12044852 A/C polymorphism between case and control groups. SPSS statistical software (version 16, SPSS Inc., Chicago, IL, USA) software was employed for calculating the mean of all of the variables in both main groups and among four classifications of disease. Analysis of variance (ANOVA) test and Chi-square test were used to compare the response of IFN-β therapy in RRMS patients harboring various rs12044852 genotypes. Hardy Weinberg equilibrium was evaluated for genotypes of rs12044852 using Pearson’s Chi-square test.

### Ethical considerations

All enrolled patients have filled the consent form to participate in this study according to the protocol of the Ethical Review Board of Tehran University of Medical Sciences.

## Results

The female/male ratio was 138/62 and 130/70 in patients and controls respectively. The mean of age was 35.38 ± 9.73 and 35.96 ± 9.87 in patients and controls respectively (Table 1). Pearson’s Chi- square and t tests did not show any significant difference in gender and age distribution between case and control groups (p>0.05, χ^2^= 0.2).

### Frequency of rs12044852 A/C polymorphism in case and control groups

**Fig 1:**
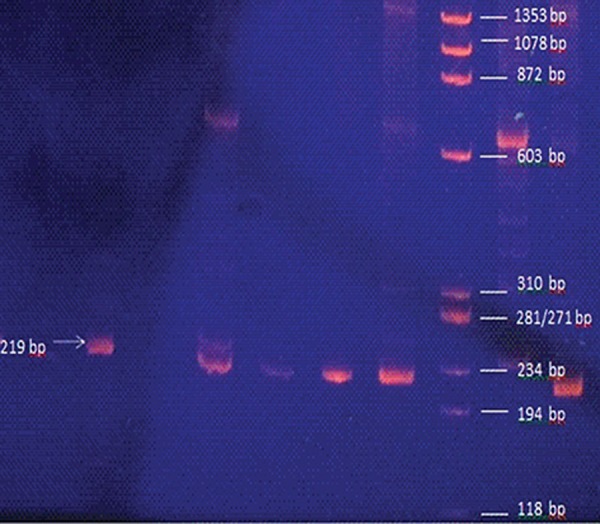
The polymerase chain reaction by specific sequence primers (PCR-SSP) products separated on acrylamide gel (10%).

A part of the PCR-SSP results is represented in figure 1. Frequency of different genotypes and alleles of rs12044852 A/C polymorphism in both case and control groups is given in table 2. Computation of expected and observed frequency of three CC, AC and AA genotypes in both MS patients (χ^2^=0.1, p=0.3, df=2) and control (χ^2^=0.7, p=0.9, df=2) groups showed that this variant was not deviated from Hardy Weinberg equilibrium in our population study. The CC and AA genotypes were the most and least frequent genotypes in both case and control groups. However, CC genotype was significantly more common in MS patients versus healthy controls (p=0.001, OR=2.22) whereas the AC genotype had meaningful higher frequency in the control group. The frequency of AA genotype was too low to be assessed by statistical means (Table 2).

**Table 2:** Distribution of rs12044852 A/C polymorphism genotypes and alleles in MS patients and controls


Frequency	Patients (n=200)	Controls (n=200)	P value	OR (CI:95%)

**Genotype frequencies**				
**CC**	167 (83.5%)	139 (69.5%)	0.001	2.22 (1.37- 3.58)
**AC**	30 (15%)	51(25.5%)	0.01	0.51 (0.31-0.85)
**AA**	3 (1.5%)	10(5%)	0.08	0.2 (0.07-1.06)
**Allele frequencies**	**Patients (n=400)**	**Controls (n=400)**	**P value**	**OR (CI:95%)**
**A**	36 (9%)	71(17.75%)	0.0003	0.4 (0.2-.07)
**C**	364 (91%)	329 (82.25%)	0.0003	2.18 (1.42-3.47)


MS; Multiple sclerosis, CI; Confidence of interval and OR; Odds ratio.

The frequency of alleles A and C was also determined in both case and control groups (Table 2). Nine percent of MS patients and 17.75% of controls had the allele A while the frequency of allele C was 91% and 82.25% in MS patients and controls respectively. There was a strong difference in the number of alleles C and A between case and control groups (p<0.0003) despite observing no significant difference between male and female MS patients harboring alleles C or A as well as CC, AC or AA genotypes. The same outcome was held for average ages and the age of onset of disease in MS patients with three genotypes and two alleles of rs12044852 polymorphism (p<0.05). The CC genotype was the most prevalent genotype in both gender groups of MS patients. ANOVA analysis demonstrated that there was no association among the average interval duration linking the first attack and relapse of disease and any genotypes of rs12044852 polymorphism of CD58 in RRMS patients.

The frequency of genotypes of this polymorphism was not associated with any of the four major subgroups of MS patients. This lack of association was also found in the assessment of the severity of MS in our enrolled patients despite a higher MSSS mean score in patients whose genotype were CC.

### Association between rs12044852 A/C polymorphism and response to IFN-β therapy

Of 200 MS patients, 131 participants suffered from RRMS. One hundred twenty RRMS patients agreed to be treated with IFN-β and were followed up under our observation for two years. Response to treatment IFN-β was evaluated using EDSS and also considering the number of episodes of attacks that occurred during the period of follow up. The EDSS scores, before (as baseline EDSS) and two years following the start of our designed treatment program, were compared to determine the response rate in genotyped RRMS patients. None of the observed patients was lost to follow up.

**Fig 2:**
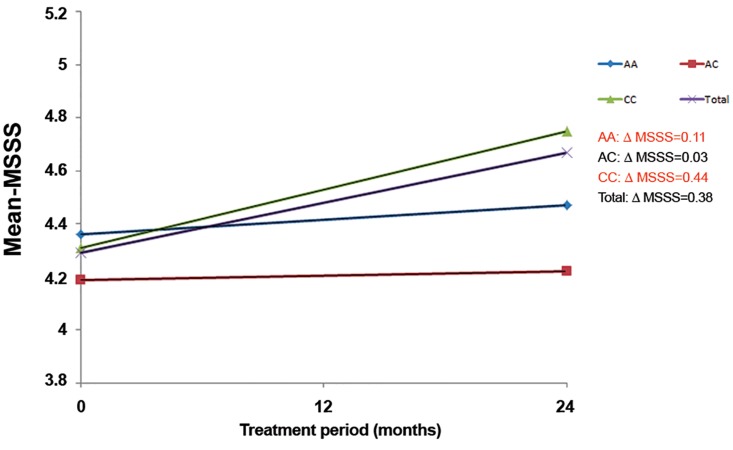
Association between response to IFN-β therapy and rs12044852 A/C polymorphism. MSSS (1); Multiple sclerosis severity score before treatment and MSSS (2); Multiple sclerosis severity score after treatment. ΔMSSS were 0.11, 0.03, 0.44 and 0.38 in MS patients with AA, AC, CC genotypes and total AD respectively. Total; All AD patients with any genotypes.

To have a more precise criteria for determining the severity of disease in response to IFN-β therapy, the mean of MSSS was also measured before and two years after initiating the treatment. The mean of MSSS was 4.36 and 4.47 in AA genotypes before and two years after treatment respectively. It was also 4.19 and 4.22 in MS patients with genotype AC whereas CC genotype patients showed the most increase in ΔMSSS during the schedule of follow up with means of 4.31 and 4.75. Among 120 RRMS patients admitted to be treated with IFN-β, 62 patients showed positive response while 58 patients had no improvement in their disease progression. It was demonstrated that the frequency of CC genotype was meaningfully higher in non-responder patients as the mean of MSSS had the most increase among others after two years of follow up (Fig 2). In contrast, RRMS patients who were carriers of AC genotype had the best response to IFN-β therapy revealed by the least ΔMSSS (0.03). In aggregate, ΔMSSS analysis exhibited association of rs12044852 polymorphism with response to IFN-β therapy in RRMS patients (p<0.05).

Moreover, it was shown here that gender had no meaningful influence on the response to IFN-β therapy for all genotypes. Therefore, there was no evidence for this polymorphism to have an influence on differential response to IFN-β therapy between male and female patients.

## Discussion

Over expression of CD58 in remission period implies a protective role for this protein against MS through inducing differentiation of regulatory T cells (Treg cells), involved in controlling the activity of auto aggressive T lymphocytes including Th17 cells (17).

The role of rs12044852 A/C polymorphism of CD58 has been confirmed as a negative effector on CD58 expression leading to down regulation of FoxP3 and consequent abnormal modulation and function of Treg cells. Recent meta-analysis on the association between this polymorphism and MS has introduced this variant as a main risk factor, in particular in Caucasians populations. However, this association was not replicated in some populations such as the African Americans (18, 19). Strong association was found in the present study based on 200 MS patients and equal number of healthy controls among genotypes and alleles of this CD58 polymorphism and susceptibility to MS disease. The OR of allele C was 2.18 was found to be more prominent than the previous study (OR: 1.24) (20). The same results were reported in several familial and case-control based association studies in MS and some other autoimmune diseases especially in recent years. The primary report was on Australian MS patients who were genotyped for rs12044852 A/C polymorphism along with additional 16 single nucleotide polymorphisms (SNPs) of other possible involved genes. Their results were consistent with ours in finding no association of rs12044852 A/C polymorphism with age of onset and patient’s gender as well as severity, progression and type of MS (21). Further confirmation of this association came from a study on 1077 Swedish MS patients compared to 10277 healthy controls which had encouraged the focus of researchers from some other populations on it (22). In this way, genotyping of Canadian, Dutch and then, New Zealand patients suffering from MS have verified the role of this variant on larger sample sizes (19, 23-26). An investigation on 43 English extensive familial aggregation and 211 sporadic cases of MS has described the C allele at rs12044852 as one of the major non-HLA risk alleles and there is merit for it to be genotyped in high risk families (23). De Jager et al. (11) confirmed the association of rs12044852 polymorphism with MS and they also demonstrated the protective effect of allele G of rs2300747 polymorphism of CD58 against MS development.

The strong association of rs12044852 was further replicated in a recent large study on 591 Dutch MS patients and 600 controls even after adjusting for multiple testing (p=0.004) (19).

Given the importance of CD58 in modulation of immune function and the replicated association of rs12044852 with MS which was confirmed again in this study, we decided to determine the impact of this variant on the efficacy of IFN-β therapy and outcome of treatment for the first time in a prospective manner. The MSSS score was used to determine the change in severity of disease due to its strong capability to compare the progression of MS over time (27). In this regard, we could find strong association after two years follow up of RRMS patients given IFN-β therapy. Both types of IFN-β including 1a and 1b are the putative interferon types prescribed for treatment of RRMS and no difference in effect was reported for them (28). Nevertheless, more than 20-55% of MS patients do not or weakly respond to them. A minor cause of this failure to respond is due to rising antibody levels against it or gene variations within the IFN-β receptors or other elements of its signaling pathway (29, 30). However, the remaining causes are presumably due to the underlying mechanisms of MS pathogenesis which determines the response to IFN-β. Several attempts have been made to reveal the role of gene expression profiles and genetic polymorphisms responsible for this variation seen in response to IFN-β (31, 32). The most critical elements of genes described to cause progression of disease in poor responders to IFN-β include glypican 5 (GPC5) and genes involved in IFN-I pathway (33). Among gene polymorphisms, combination of variants within the non-HLA JAK2, IL10RB, GBP1, PIAS, IL10 genes and some others as well as HLA genes have shown significant association with non-responding status of MS patients to IFN-β therapy (34-36). Given the up regulation of CD58 in response to IFN-β therapy (12), it seems that rs12044852 may interfere in the expression of this gene leading to the best response to IFN-β therapy

Give the significant association found between rs12044852 polymorphism and response to IFN-β therapy, genotyping of MS patients before starting the treatment plan may have critical effects on survival and patient’s outcome. However, unctional analysis at cellular and animal models is warranted to reveal how this intronic polymorphism affects the activity of CD58 protein. Elucidating and confirming the effect of rs12044852 polymorphism on efficiency of IFN-β therapy in larger sample sizes could open the window toward personalized medicine to targeted treatment and improvement of the half life of MS patients.

## Conclusion

Astrong association was found between rs12044852 and MS. Its effect on response to treatment with IFN-β in RRMS patients was also significant. Further studies are warranted to clarify the molecular pathology of this polymorphism responsible for worse prognosis of MS patients especially in response to IFN-β therapy.
